# Genetic variation in FOXP3 and ROR-γ genes in pediatric acute lymphocytic leukemia (ALL) patients: correlation with associated cytokines

**DOI:** 10.1007/s12672-022-00549-3

**Published:** 2022-09-09

**Authors:** Eman A. El-maadawy, Rania M. Bakry, Mohamed M. Moussa, Sobhy Hasab El-Naby, Roba M. Talaat

**Affiliations:** 1 Molecular Biology Department, Genetic Engineering and Biotechnology Research Institute (GEBRI], University of Sadat City, El Sadat City, Egypt; 2 South Egypt Cancer Institute, Assiut University, Assiut, Egypt; 3 Clinical Hematology and Bone Marrow Transplantation, Ain-Shams University, Cairo, Egypt; 4 Zoology Department, Faculty of Science, Menoufiya University, Menoufiya, Egypt

**Keywords:** Pediatric ALL, *FOXP3*, ROR-γ, SNPs, Cytokines, Treg/Th17 cells

## Abstract

**Background:**

*FOXP3* and *ROR-γ* genes are master regulators of the Treg and Th17 differentiation, respectively. This work was planned to investigate the impact of *FOXP3* (rs3761548C/A and rs3761549C/T) and *ROR-γ* (rs9017A/G & rs9826A/G) gene polymorphism on the vulnerability of pediatric Egyptians to acute lymphoblastic leukemia (ALL). Furthermore, we evaluated the impact of these genetic variations on Treg/Th17-related cytokines.

**Methods:**

*FOXP3* SNPs were genotyped using PCR-based restriction fragment length polymorphism (PCR-RFLP), while *ROR-γ* SNPs polymorphism were performed by PCR-sequence-specific primer (PCR-SSP)**.** An Enzyme-linked immunosorbent assay (ELISA) was used to assess the levels of Treg/Th17 associated cytokines on 128 ALL children and 124 healthy donors.

**Results:**

Compared to controls, patients had a significant increase (p < 0.01/p < 0.05) in *FOXP3*rs3761548CC genotype and a significant decrease (p < 0.001/p < 0.01) inrs3761548CA genotype. A significant elevation (p < 0.001/p < 0.01) in *ROR-γ* rs9017AA genotype and a significant reduction (p < 0.01/p < 0.05) in rs9017AG genotype were detected in ALL patients versus controls. An insignificant change in *FOXP3* (rs3761549C/T) and *ROR-γ* (rs9826A/G) genotypes was demonstrated between both groups. *ROR-γ* GG and GA haplotypes were significantly decreased (p < 0.05/p < 0.05; p < 0.05/p < 0.05) in ALL subjects compared to healthy ones. Relapsed patients had a significantly higher (p < 0.05/P < 0.05) frequency of *FOXP3* rs3761548CA genotype than non-relapsed subjects. *ROR-γ* rs9017AG and rs9826GG genotypes might be associated with the increase in IL-23 plasma level.

**Conclusions:**

Our preliminary data provided evidence for the impact of*FOXP3* (rs3761548C/A) and *ROR-γ* (rs9017A/G) gene polymorphisms and the occurrence of ALL in Egyptian children. Another large-scale prospective study should be conducted to validate these findings.

## Background

Leukemia is a malignant disease that affects the proliferation and growth of leukocytes and their precursors over time [1]. The most common form of leukemia in children is acute lymphoblastic leukemia (ALL), with an 80% cure rate [2]. It accounts for 25–30% of cancers in children and adolescents aged 0 to 18 years [3]. Clinical evidence has recently highlighted the role of CD4 + T cells in the development and maintenance of effective anti-tumor immunity [4].

CD4^+^T cells can be divided into several subgroups, including Th1, Th2, Th17, Treg, Th9, and Th22[5]. Treg/Th17 balance is essential for maintaining the homeostasis of anti-tumor immunity [6]. Recently, it has become widely accepted that alteration in the equilibrium between Treg and Th17 cells is associated with several types of cancers [7]. Tregs are correlated with cancer progression, while Th17 cells have been implicated in carcinogenesis, although; their role is less understood [5]. The differentiation of Th17 cells and Tregs is controlled by a combination of cytokine milieu, transcriptional activities, and genetic control [7]. Moreover, several cytokines exert profound effects on the progression of hematopoietic malignancies [8].

Forkhead box P3 (*FOXP3*), the Treg's signature transcriptional activator, is essential for Treg development and function [9]. The *FOXP3* gene, with 11 encoding exons and three non-coding exons, is located on the X-chromosome (Xp11.23) [10]. Single nucleotide polymorphisms (SNPs) in the *FOXP3* gene can affect its expression and impair Tregs' suppressive function [11]. The promoter region of the *FOXP3* gene contains five SNPs; among them, the rs3761548C/A and rs3761549C/T gene, with have been implicated to be associated with cancer risk [11, 12].

Retinoic acid receptor-related Orphan Receptor gamma (RORγ or RORc) encodes RORγt, the master transcription factor expressed by Th17 cells [13]. The *ROR-γ* gene, more than 21 kb, is mapped to human chromosome 1q21.3 and comprises711 exons separated by ten introns [14, 15]. The promoter region of *ROR-γ* was identified to have several elements required for promoter activity and gene expression in Th17 [16]. Little is known regarding the SNPs in the *ROR-γ* gene. Only two SNPs, rs9017 G/A and rs9826 A/G (situated in the 3' UTR), were investigated in rheumatoid arthritis in the Polish population [17] and acute kidney injury development in Iranian patients [18].

Since Treg/Th17 cells play a vital role in cancer, the current study is designated to illuminate the potential genetic link between *FOXP3* (rs3761548C/A and rs3761549C/T) and *ROR-γ* (rs9017A/G & rs9826A/G) SNPs and the occurrence of pediatric ALL in the Egyptian population insights to the clinical disease manifestation. In addition, we investigate the gene-gene interaction between both genes to understand their cumulative effect on the disease better. Furthermore, we investigate the impact of these SNPs on the level of Treg-related cytokines (Interleukin (IL)-10 and transforming growth factor (TGF)-β) and Th-17-related cytokines (IL-6, IL-17, IL-23). This study is the first report of *ROR-γ* gene polymorphisms in pediatric ALL that we are aware of.

## Subjects and methods

### Patients and controls

A total of 128 ALL children(44males and 84females; mean age of 7.18 ± 0.38 years)were enrolled sequentially from the pediatric oncology department at Assiut University's South Egypt Cancer Institute and Hospital, Egypt. Patients were classified based on the disease's severity [19].The medical oncologist recorded hematological, biochemical, and immunophenotyping data from patients' reports during follow-up. All patients were treated based on the total XIIIB regimen [20]. The patient is deemed to be responder when its peripheral haemgraph starts to normalize along with the disappearance of lymphoblasts from peripheral blood and elimination (less than 5%) in the bone marrow. At diagnosis, count of white blood cell (WBCs), age, and bone marrow smears were the main factors used to determine disease risk. According to risk classification, ALL children were categorized as low risk: 1–10 years old with a WBCs count of < 50,000/mm^3^ and moderate/high risk:: < 1 and > 10 years old with a WBCs count > 50,000/mm^3^. Overall survival rate of ALL patients was recorded based on the period of the patients that can be lived after starting treatment. Relapse refers to the return of ALL in patients who have already undergone treatment for the disease. The control group consisted of approximately 124 age- and sex-matched donors who were free of chronic diseases had no family history of leukemia, lived in the same geographic region, and shared the same ethnic background as the patients.

#### Exclusion criteria

Patients older than 18 years or who received chemotherapy outside South Egypt Cancer Institute or received corticosteroid therapy before or during chemotherapy or with an interrupted chemotherapy regimen were excluded from this study.

#### Ethical approval

All procedures involving human subjects in our study were carried out in compliance with the guidelines for clinical research established by the Human Ethical Clearance Committee in accordance with the Helsinki Declaration (1964) and with the consent of the human subject. South Egypt Cancer Institute and Hospital, Assiut University's Ethical Clearance Committee (http://www.aun.edu.eg/faculty-medicine/EthicalCommittee.php) approved the study (IORG: IORG0006563 and IRB: IRB00007877).

#### Informed consent

Until blood collection, each patient's informed consent was obtained from a parent or legal guardian, and the consent procedure was approved by the ethics committee/institutional review board.

### DNA isolation

Three mL of venous blood was obtained from each participant and placed in an ethylene-diamine-tetra acetic acid (EDTA) sterile tube by vane puncture. For cytokine analysis, the tubes were centrifuged at 1500 rpm for 10 min, and the plasma was isolated and stored at −80 °C. According to protocol, genomic DNA was extracted from whole blood samples using the GentraPuregene Blood Kit (Qiagen Business, Hilden, Germany). The extracted DNA was electrophoresed on a 1% agarose gel to ensure DNA integrity. The NanoDrop™ 2000/2000c Spectrophotometer (Thermo Fisher Scientific, Waltham, MA, USA) was used to determine the purity and concentration of DNA.

### Genotyping of *FOXP3* and *ROR-γ*

The 2720 thermal cycler conducted all polymerase chain reactions (PCR) (Applied Biosystems). As previously defined, PCR-based restriction fragment length polymorphism (PCR–RFLP) was used to genotype *FOXP3* (rs3761548C/A and rs3761549C/T) [21]. ROR- γ (rs9017A/G & rs9826A/G), on the other hand, was genotyped using PCR- sequence-specific primers (PCR-SSP). Primosnp 3.4 (http://www.changbioscience.com/primo/ primosnp.html) was used to design the primer sequences, which were then confirmed by primer blast (http://www.ncbi.nlm.nih.gov/tools/primer-blast). Primers used for genotyping *OR-γ* (rs9017A/G) were: Forward A: 5'-GCTGTGAACCCTCCCCTGAA-3', Forward G: 5'-GCTGTGAACCCTCCCCTGAG-3' and Reverse:5'-GTGAGGGTGGGTTGGATCTG-3' while Forward A:5'-CGCACTGGTCAGTCGGAA-3', Forward G:5'- CGCACTGGTCAGTCGGAG-3' and Reverse: 5'- GTGAGGGTGGGTTGGATCTG -3' were used for rs9826A/G SNP.

For each SNP, the PCR reaction was performed in two tubes, one for each allele, with a final reaction volume of 25 μl. In the PCR mixtures, 2 × DreamTaq Green Master Mix (Fermentas, Thermo Fisher Scientific Inc.), 1 pmol of each allele-specific primer, 10 pmol of reverse primer, and 200 ng of DNA template was used. The following were the PCR cycling conditions for *ROR-γ* (rs9017A/G): 94 °C for 5 min [1 cycle], then 94 °C for 30 s, 62 °C for 30 s, and 72 °C for 60 s [30 cycles], with a final extension stage at 72 °C for 7 min. For *ROR-γ* (rs9826A/G), the amplification conditions were as follows: One cycle of 5 min at 94 °C followed by 30 cycles of denaturing for 30 s at 94 °C, annealing for 30 s at 68 °C, and extension for 30 s at 72 °C and finally, a final extension step at 72 °C for 7 min.

The size of the PCR product was 296 bp and 510 bp for *ROR-γ* rs9017A/G andrs9826A/G, respectively. A 2% agarose gel electrophoresis was used to visualize the PCR products. The size of PCR products was estimated compared to a 100 bp DNA ladder (Fermentas). Since PCR-SSP can easily produce a false-positive result, 20% of samples were selected at random and genotyped twice to ensure genotyping accuracy and to confirm our findings.

### Cytokine evaluation

IL-10, TGF-β, IL-6, IL-17, and IL-23 cytokine levels were tested in the plasma of patients and healthy subjects utilizing DuoSet sandwich enzyme-linked immunosorbent assay (ELISA) development kits (R&D Systems, Inc., Minneapolis, MN, USA) according to the manufacturer's guidelines. The intensity of the formed color was assessed using a microplate reader (Sunrise; Tecan Group Ltd, Männedorf, Switzerland) to read optical absorbance at 450 nm. The digital data of raw absorbance values were processed into a standard curve by the ELISA reader-controlling program (Softmax; Molecular Devices, Sunnyvale, CA, USA), from which the cytokine concentrations of the specimen were calculated. Results were expressed as a picogram of cytokine per milliliter plasma (pg/ml).

### Statistical analysis

The Statistical Package for Social Science (SPSS) version 21 was used to analyze statistical data (IBM Corporation, USA). Patients and control groups were compared utilizing an independent T-test, and the results were displayed as the mean ± standard error. Categorical data were given as frequencies (%). Allele, genotype, and haplotype distributions were compared using Chi-squared (X^2^) tests. Furthermore, the relative risks in both control and ALL patients were calculated using the odds ratio (OR) and 95% confidence intervals (CI). Spearman's correlation test was used to determine the correlation between various variables. Haplotype reconstruction and Linkage Disequilibrium (LD) parameters (D′ and r^2^) from population genotype statistics were manipulated by SNPstats, an online tool (https://www.snpstats.net/start.htm).The prospective high dimensional gene–gene interactions between SNPs were identified using the Multifactor dimensionality reduction (MDR) software version3.0.2 (http://sourceforge.net/projects/mdr/).The overall survival (OS) was calculated as the time from diagnosis to fatality or last contact. Probabilities of OS and proportional hazards of relapse were estimated using the Kaplan–Meier method [22]. The log-rank test was used to determine differences between survival curves. All results were two-tailed, and P values of less than 0.05 were deemed statistically significant and was corrected using Bonferroni correction.

## Results

### Subjects characteristics

Comprehensive demographic, hematological, biochemical data and clinical characteristics of all subjects were displayed in Table 1. Most patients were at low risk of age and WBCs, with 76.6% of cases responding to treatment and around 20.4% relapsing.

**Table 1:** Demographic, biochemical and hematological, characteristics of ALL patients and healthy controls

Laboratory investigations	Control group (N = 124) (Mean ± SE)	ALL group (N = 128) (Mean ± SE)	P
Demographic data			
Age (year)	6.67 ± 0.30	7.18 ± 0.38	NS
Gender (M/ F)	58/66	44/84	NS
Biochemical Data			
AST (IU/L)	25.16 ± 0.60	43.65 ± 5.71	**P < 0.01**
ALT (IU/L)	22.56 ± 0.45	32.24 ± 3.72	**P < 0.01**
ALB (g/L)	43.72 ± 0.41	36.00 ± 1.17	**P < 0.001**
Bilirubin (mg/dl)	0.66 ± 0.01	0.86 ± 0.25	NS
Urea (mg/dl)	24.33 ± 0.75	38.98 ± 15.56	NS
Creatinine (mg/dl)	0.35 ± 0.01	0.61 ± 0.16	NS
LDH (IU/L)	305.52 ± 4.44	1803.71 ± 217.98	**P < 0.001**
Hematological Data			
WBCs (10^3^/µL)	8.04 ± 0.18	46.53 ± 6.60	**P < 0.001**
Hemoglobin (g/dl)	11.49 ± 0.11	8.15 ± 0.21	**P < 0.001**
PLT (10^3^/µL)	316.76 ± 5.94	81.60 ± 7.40	**P < 0.001**
Clinical data			
Low risk < 50,000/Ml		89 (69.5%)	
High risk > 50,000/Ml		39 (30.5%)	
Age at diagnosis			
Low risk 1–10 years		75 (58.6%)	
high risk > 10 years		53 (41.4%)	
Response for treatment			
Responders		98 (76.6%)	
Non responders		30 (23.4%)	
Immunophenotype			
B-ALL		107 (83.6%)	
T-ALL		21 (16.4%)	
Relapse			
No relapse		102 (79.6%)	
Relapsed		26 (20.4%)	
Outcome			
Alive		118 (92.2%)	
Dead		10 (7.8%)	
CNS II			
Yes		83 (72.8%)	
No		31 (27.2%)	
CNS III			
Yes		42 (40.8%)	
No		61 (59.2%)	

All data are presented as mean ± SE

Bold refereed to significant values

*WBCs* White blood cells, *PLT* Platelet, *AST* Aspartate aminotransferase, *ALT* Alanine aminotransferase, *ALB* Albumin, *IU/L* International Units per liter, *g/L* Gram per liter, *mg/dL* Milligrams per Deciliter, *mEq/L* Milliequivalents Per Litre, *NS* not significant

### Association between *FOXP3/ROR-γ* polymorphism and ALL

The distribution of genotypes/alleles frequency of *FOXP3*SNPs (rs3761548C/A andrs3761549C/T) in both pediatric ALL and control groups were shown in Table 2. The rs3761548C/A polymorphism in *FOXP3*revealed a significant increase (p < 0.01/p < 0.05) in the rs3761548CC genotype and a significant decrease (p < 0.001/p < 0.01) in the rs3761548CA genotype in female ALL children compared to female controls. In terms of allelic frequency, there was no statistically significant variation between both groups. The *FOXP3* rs3761548CC (OR = 3.07, 95%CI 1.40–6.73, p < 0.01/p < 0.05) and AA (OR = 1.66, 95%CI 0.78–3.52, p > 0.05) genotypes could be associated with an increased ALL risk. However, there was no significant change in the distribution of all genotypes/alleles between the patient and control groups when the *FOXP3* rs3761549C/T SNP was examined.

**Table 2:** Genotype distribution and allelic frequency of the FOXP3 SNPs (rs3761549C/T,and rs3761548C/A) in controls and ALL patients

**Position**	**Control group** **(N = 124)**	**ALL group** **(N = 128)**	**OR (95% CI)**	**P/pc**
rs3761548 C/A (N,%)				
	Female (66)	Female (84)		
C/C	11 (17%)	32 (38%)	**3.07 (1.40–6.73)**	**P < 0.01/p < 0.05**
C/A	41 (62%)	26 (31%)	0.27 (0.13–0.53)	**P < 0.001/p < 0.01**
A/A	14 (21%)	26 (31%)	**1.66 (0.78–3.52)**	NS
CA/AA	55 (83%)	52 (74%)	0.32 (0.14–0.71)	**P < 0.01/p < 0.05**
C	63 (48%)	90 (54%)	1.26 (0.80–1.99)	NS
A	69 (52%)	78 (46%)	0.791 (0.50–1.24)	NS
	Male (58)	Male (44)		
C	73 (63%)	58 (66%)	1.13 (0.63–2.03)	NS
A	43 (37%)	30 (34%)	0.87 (0.49–1.56)	NS
rs3761549C/T (N,%)				
	Female (66)	Female (84)		
C/C	20 (30%)	21 (25%)	0.76 (0.37–1.57)	NS
C/T	34 (52%)	51 (61%)	1.45 (0.75–2.79)	NS
T/T	12 (18%)	12 (14%)	0.75 (0.31–1.79)	NS
CT/TT	46 (70%)	63 (67%)	1.30 (0.63–2.68)	NS
C	74 (56%)	93 (55%)	0.97 (0.61–1.53)	NS
T	58 (44%)	75 (45%)	1.02 (0.65–1.62)	NS
	Male (58)	Male (44)		
C	61 (53%)	51 (58%)	1.26 (0.72–2.20)	NS
T	55 (47%)	37 (42%)	0.79 (0.45–1.37)	NS

Bold refereed to significant values

*P* p-value (significant), *Pc* p-value corrected, *NS* Not significant

*ROR-γ* (rs9017A/G & rs9826A/G) genotypes and allelic frequencies were shown in Table 3 Analysis of *ROR-γ* (rs9017A/G) revealed that the AG genotype was the highest frequency genotype, followed by AA and GG genotypes in both groups. When compared to controls, ALL patients had a significant increase (p < 0.001/p < 0.01) in rs9017AA genotype with a significant reduction (p < 0.01/p < 0.05) in AG genotype. Furthermore, the A allele was more frequent in both groups than the G allele. Comparing ALL patients to controls, the rs9017A allele was significantly increased (p < 0.05/ p < 0.05). In contrast, the G allele was significantly decreased (p < 0.05/p < 0.05). rs9017AA genotype (OR = 3.31, 95%CI 1.69–6.50, p < 0.001/p < 0.01) and A allele (OR = 1.56, 95%CI 1.09–2.23, p < 0.05/p < 0.05) might be considered risk factors, while AG (OR = 0.42, 95%CI 0.23–0.77, P < 0.01/P < 0.05), GG (OR = 0.31, 95%CI 0.06–1.57, p > 0.05) genotypes, and G allele (OR = 0.63, 95%CI 0.44–0.91, p < 0.05/NS) might be deemed protective. Regarding *ROR-γ* (rs9826A/G), the AG genotype was the most common, followed big and AA genotypes. There was no significant difference in the distribution of genotypes or alleles among normal subjects and ALL patients. The GA genotype (OR = 1.33, CI 0.71–2.46, p > 0.05) is deemed a risk factor for pediatric ALL, while AA and GG genotypes (OR = 0.62, 95%CI 0.21–1.82, p > 0.05; OR = 0.90, 95%CI 0.44–1.82, p > 0.05; respectively) might be protective.

**Table 3:** Genotype distribution and allelic frequency of ROR-γ (rs9017A/G and rs9826A/G) in controls and ALL patients

Cytokine gene	Control group (N = 124)	ALL group (N = 128)	OR (95% CI)	P/pc
ROR-γ (rs9017A/G) Genotype frequency (N, %)				
A/A	14 (11%)	38 (29%)	**3.31 (1.69–6.50)**	**P < 0.001/p < 0.01**
A/G	104 (84%)	88 (69%)	0.42 (0.23**–**0.77)	**P < 0.01/p < 0.05**
G/G	6 (5%)	2 (2%)	0.31 (0.06**–**1.57)	NS
AG/GG	110 (89%)	90 (70%)	0.30 (0.15–0.59)	**P < 0.001/p < 0.01**
Allele Frequency (N, %)				
A	132 (53%)	164 (64%)	**1.56 (1.09–2.23)**	**P < 0.05/ P < 0.05**
G	116 (47%)	92 (36%)	0.63 (0.44–0.91)	**P < 0.05/ P < 0.05**
ROR-γ (rs9826A/G) Genotype Frequency (N, %)				
A/A	9 (7%)	6 (5%)	0.62 (0.21–1.82)	NS
A/G	96 (77%)	105 (82%)	1.33 (0.71–2.46)	NS
G/G	19 (16%)	17 (13%)	0.90 (0.44–1.82)	NS
AG/GG	115 (93%)	122 (95%)	**1.59 (0.54**–**4.61)**	NS
A	114 (46%)	117 (46%)	0.93 (0.66–1.33)	NS
G	134 (54%)	139 (54%)	1.06 (0.74–1.51)	NS

Bold refereed to significant values

*P* p-value (significant), *Pc* p-value corrected, *NS* Not significant

Table 4 described the haplotype frequencies of the *FOXP3* (rs3761549C/T and rs3761548C/A) and *ROR-γ* (rs9017 A/G & rs9826 A/G) SNPs in ALL patients and controls. The estimates of haplotype frequencies revealed four haplotypes for *FOXP3* SNPs: CC, CT, AC, and AT. No statistically significant variation in haplotype distribution was found between the two groups/genders. Regarding LD, the haplotype estimates for males (D' = 0.053, r^2^ = 0.001) and females (D' = 0.085, r^2^ = 0.006) indicated that there was no LD between *FOXP3*both SNPs (rs3761549C/T and rs3761548C/A) in the Egyptian children. On the other hand, four haplotypes had emerged (AA, AG, AG, and GA) for the *ROR-γ* Sensate AA haplotype was the most frequent in patients and GG in controls. The GA haplotype in both groups was the lowest. When patients were compared to controls, there was a significant reduction in GG (p < 0.05/p < 0.05) and GA (p < 0.05/p < 0.05) haplotype. The OR values revealed that no haplotype was a disease risk factor. The LD pattern between *ROR-γ* both SNPs (rs9017A/G and rs9826A/G) demonstrated a significant LD (p < 0.001/p < 0.01), with a D' value of 0.763 and an r^2^ value of 0.345, suggesting a degree of LD between the two Spoof *ROR-γ* in the Egyptian population.

**Table 4:** Haplotype frequencies of the FOXP3 SNPs (rs3761549C/T and rs3761548C/A) and ROR-γ (rs9017A/G and rs9826A/G) in controls and ALL patients

Haplotype	Gender	Control group	Patients group	OR (95% CI)	P
FOXP3 (rs3761549C/T and rs3761548C/A)					
CC	Female	25.2%	33.6%	1.00	–
	male	32.2%	41.5%	1.00	–
CT	female	22.4%	21.7%	0.52 (0.24–1.14)	NS
	male	30.7%	24.3%	0.58 (0.28–1.20)	NS
AT	female	21.4%	24.6%	0.91 (0.47–1.76)	NS
	Male	16.7%	16.3%	0.70 (0.35–1.39)	NS
AC	Female	30.7%	19.9%	0.66 (0.28–1.55)	NS
	Male	20.3%	17.7%	0.79 (0.40–1.56)	NS
ROR-γ (rs9017A/G and rs9826A/G)					
AA		36.4%	43.7%	1.00	–
GG		40.2%	34.0%	0.36 (0.16–0.82)	**P < 0.05/ P < 0.05**
AG		13.8%	20.3%	0.96 (0.49–1.87)	NS
GA		6.5%	1.9%	0.15 (0.03–0.64)	**P < 0.05/ P < 0.05**

Bold refereed to significant values

*P* p-value (significant), *Pc* p-value corrected, *NS* Not significant

**Fig. 1:**
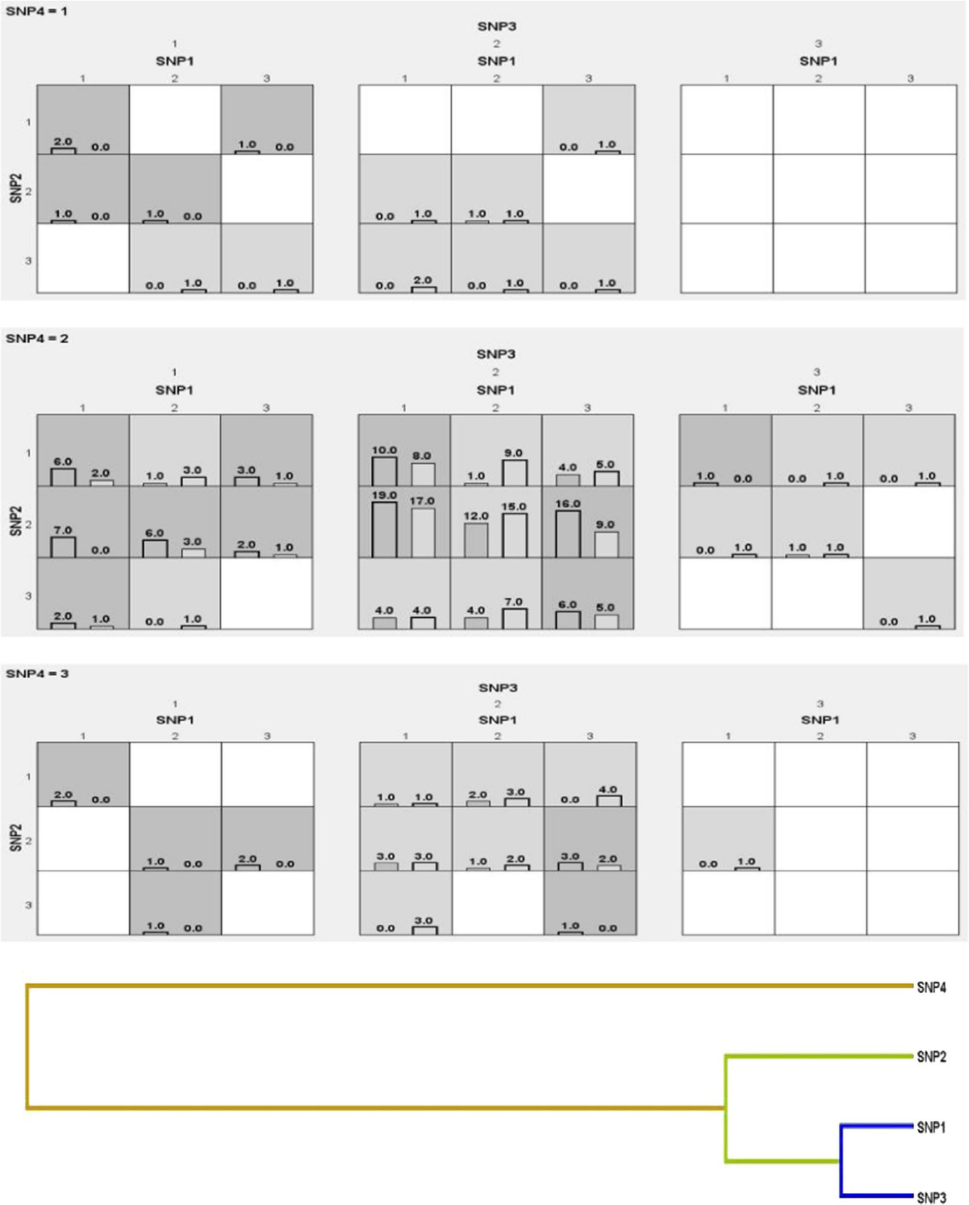
Gene-gene interaction between the 4 SNPs. SNP1;FOXP3 (rs3761549C/T), SNP2;FOXP3(rs3761548C/A), SNP3; ROR-γ (rs9017 A/G),SNP4; ROR-γ (rs9826 A/G). Dark Boxes describe the high risk for ALL versus control between the three genotypes of the four SNPs. The lightly shaded box showed low-risk combinations

All potential one-to-three-way SNP-SNP interactions in ALL patients and healthy subjects were identified based on the MDR findings. According to the results of the study, the best-elected classifiers of ALL status depending on the selected four SNP loci using the cross-validation consistency; training and assessment accuracy were as follows: single locus *ROR-γ* (rs9017 A/G) followed by *FOXP3* (rs3761549C/T) and *FOXP3*(rs3761548C/A), followed by *FOXP3* (rs3761549C/T) and *FOXP3*(rs3761548C/A) and *ROR-γ* (rs9017 A/G), followed by the four Sensate three loci consist of SNPs *FOXP3* (rs3761549C/T),*FOXP3* (rs3761548C/A), and *ROR-γ* (rs9017 A/G),and the four tested loci seem to be the best classifier cancer status of ALL. The three SNPs had training (0.6557) and testing accuracy (0.5825), while the four SNPs had 0.6693 and 0.5185, respectively, with a cross-validation consistency of 10/10 for both and higher significance with p < 0.001/p < 0.01(Fig. 1).

### Association between ALL clinical manifestations and *FOXP3*/*ROR-γ* SNPs

The Association of *FOXP3*/*ROR-γ* gene polymorphisms and the most common clinical manifestations of ALL patients were demonstrated in Table 5. Regarding *FOXP3* (rs3761548C/A),rs3761548CC genotype was significantly increased (p < 0.05/NS), and rs3761548CA genotype was significantly decreased (p < 0.001/p < 0.01) in patients' high age risk compared to those with low risk. Moreover, a significant reduction (p < 0.05/p < 0.05) rs3761548CA genotype was detected in relapsed patients compared to non-relapsed ones. Analysis of *FOXP3* (rs3761549C/T) SNP revealed a diminution (p < 0.05/NS) in rs3761549CC genotype in treatment responders compared to non-responders. In addition, rs3761549CC genotype showed an increase (p < 0.05/NS) in relapsed patients versus non-relapsed group. Both *ROR-γ* (rs9017A/G & rs9826A/G) SNPs showed no statistically significant association with ALL clinical manifestations.

**Table 5:** Association of the most common clinical findings of the disease and FOXP3&ROR-γ gene polymorphisms inALL patients

	WBCs –risk		Age risk		Response		ALL-Type		Relapse		CNSII		CNSIII	
	Low	High	Low	High	Yes	No	B-cell	T-cell	Yes	No	Yes	No	Yes	No
	83	35	75	53	98	30	107	21	26	102	83	31	42	61
FOXP3 (rs3761548C/A)														
CC	38 45.8%	16 45.7%	28 37.3%	30* **56.6%**	44 44.9%	13 43.3%	46 43.0%	12 57.1%	12 46.2%	46 45.1	36 43.4%	16 51.6%	19 45.2%	30 49.2%
CA	20 24.1%	8 22.9%	28*** **37.3%**	4 7.5%	21 21.4%	12 40.0%	29 27.1%	3 14.3%	12* **46.2%**	20 19.6%	21 25.3%	8 25.8%	11 26.2%	14 23.0%
AA	25 30.1%	11 31.4%	19 25.3%	19 35.8%	33 33.7%	5 16.7%	32 29.9%	6 28.6%	2 7.7%	36** 35.3%	26 31.3%	7 22.6%	12 28.6%	17 27.9%
FOXP3 (rs3761549C/T)														
CC	18 21.7%	11 31.4%	24 32%	10 18.9%	21* **21.4%**	13 43.3%	26 24.3%	8 38.1%	12* **46.2%**	22 21.6%	23 27.7%	6 19.4%	13 31.0%	10 16.4%
CT	53 63.9%	18 51.4%	40 53.3%	36 67.9%	62 63.3%	14 46.7%	66 61.7%	10 47.6%	12 46.2%	64 62.7%	48 57.8%	20 64.5%	22 52.4%	42 68.9%
TT	12 14.5%	6 17.1%	11 14.7%	7 13.2%	15 15.3%	3 10.0%	15 14.0%	3 14.3%	2 7.7%	16 15.7%	12 14.5%	5 16.1%	7 16.7%	9 14.8%
ROR-γ (rs9017 A/G)														
AA	22 26.5%	13 37.1%	20 26.7%	18 34.0%	30 30.6%	9 30.0%	29 21.7%	9 42.9%	8 30.8%	30 29.4%	20 21.4%	11 35.5%	12 28.6%	12 19.7%
AG	61 73.5%	21 60.0%	54 72.0%	34 64.2%	67 68.4%	21 70.0%	76 71.0%	12 57.1%	17 65.4%	71 69.6%	63 75.9%	20 64.5%	30 71.4%	49 80.3%
GG	0 0%	1 2.9%	1 1.3%	1 1.9%	1 0.8%	0 0%	2 1.9%	0 0%	1 3.8%	1 1%	–	–-	–	–
ROR-γ (rs 9826 A/G)														
AA	1 1.2%	3 8.6%	4 5.3%	2 3.8%	5 5.1%	2 6.6%	6 5.6%	0 0%	1 3.8%	5 4.9%	2 2.4%	3 9.7%	3 7.1%	0 0%
AG	70 84.3%	29 82.9%	62 82.7%	43 81.1%	81 82.7%	23 76.7%	84 78.5%	21* 100%	20 76.9%	65 83.3%	70 84.3%	24 77.4%	34 81.0%	54 88.5%
GG	12 14.5%	3 8.6%	9 12.0%	8 15.1%	12 12.2%	5 16.7%	17 15.9%	0 0%	5 19.2%	12 11.8%	11 13.3%	4 12.9%	5 11.9%	7 11.5%

Bold refereed to significant values

*P < 0.05, **P < 0.01, ***P < 0.001

**Fig. 2:**
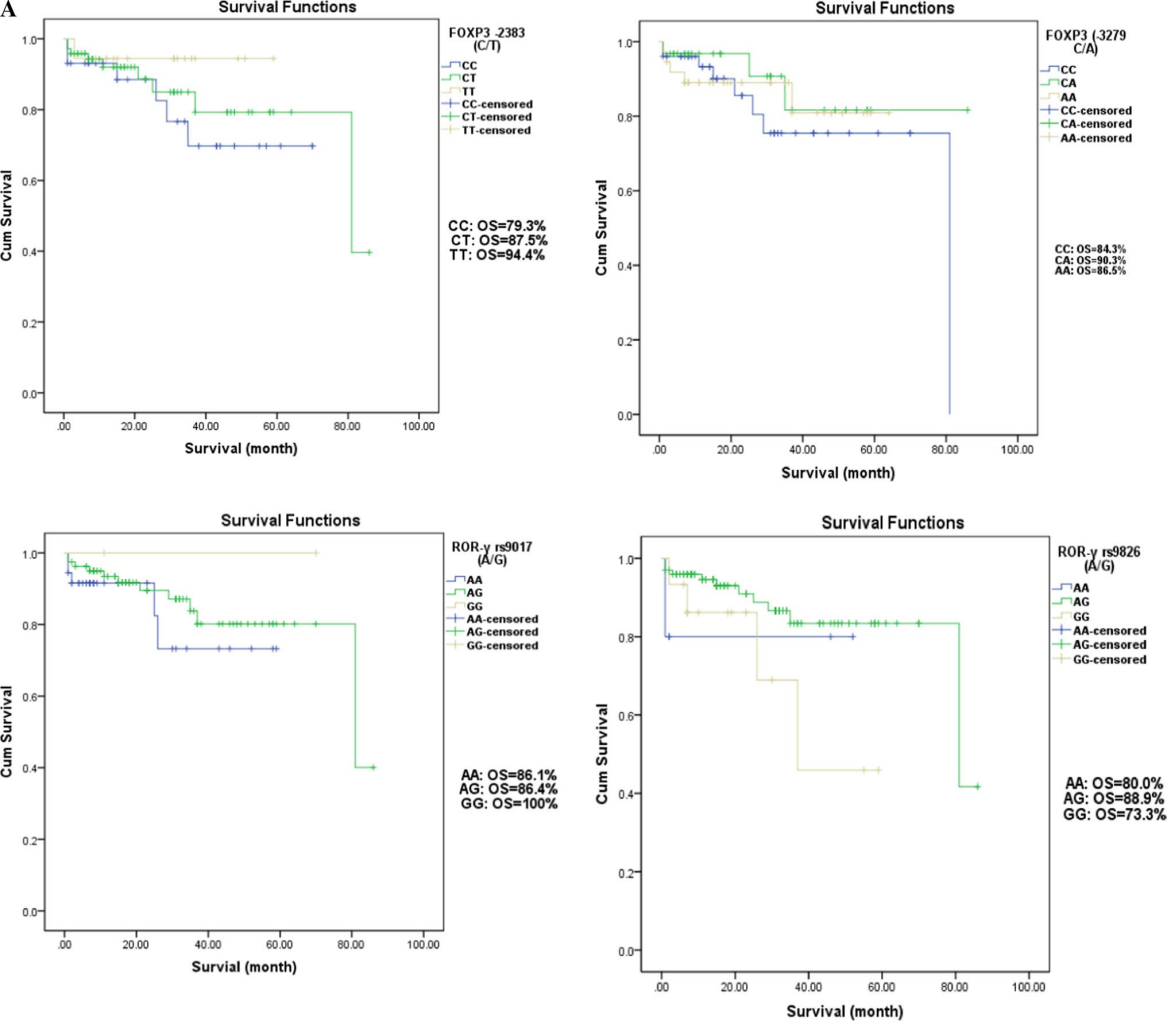

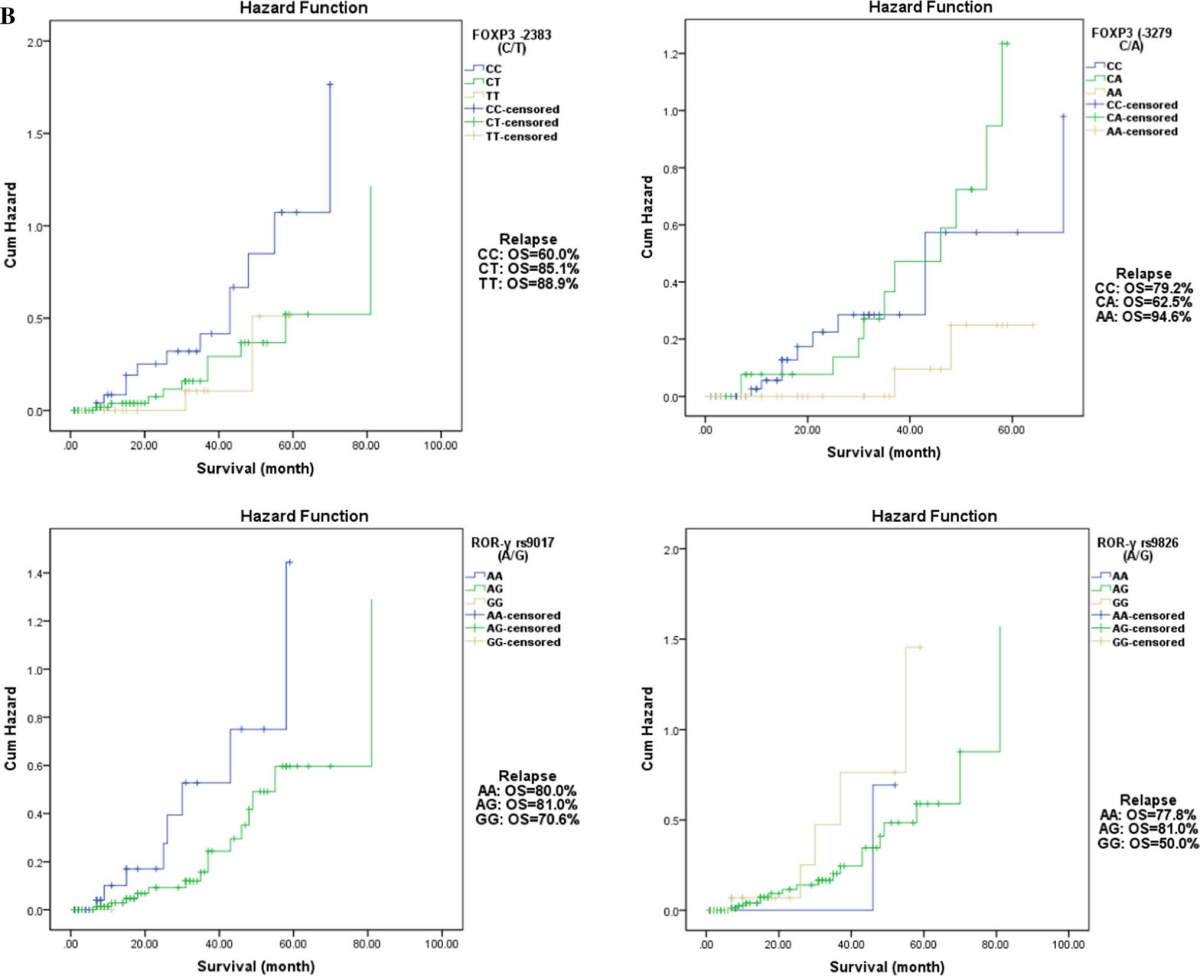
**a**. Overall Survival according to FOXP3 (−2383 (rs3761549) C/T and −3279 (rs3761548) C/A) and ROR-γ (rs9017 A/G and - rs9826 A/G) SNPs in Pediatric ALL. **b**. Disease-Free Survival According to FOXP3 (−2383 (rs3761549) C/T and -3279 (rs3761548) C/A) and ROR-γ (rs9017 A/G and - rs9826 A/G) SNPs in Pediatric ALL

To investigate whether the polymorphisms of the four SNPs of *FOXP3* (rs3761549C/T and rs3761548C/A) and *ROR-γ* (rs9017A/G & rs9826A/G) genes were associated with OS of ALL patients, the survival analyses of various genotypes are presented in Fig. 2a, b. Results pointed out that OS did not differ significantly between all studied genotypes in pediatric ALL.

### Correlation between *FOXP3*/*ROR-γ* SNPs and cytokine secretion levels

As previously mentioned by El-maadawy et al. [23], Concerning IL-10 and TGF-β levels (Treg cytokines), ALL patients had a significant decrease (p < 0.001/p < 0.01) in TGF-β level and an increase (p < 0.05/NS) in IL-10 plasma level compared to healthy subjects. Looking at Th17-related cytokines, ALL patients were found to have an insignificant reduction inIL-17 level and significant elevation in IL-23 and IL-6 (p < 0.05/NS, p < 0.01/p < 0.05; respectively) compared to normal subjects.

**Fig. 3:**
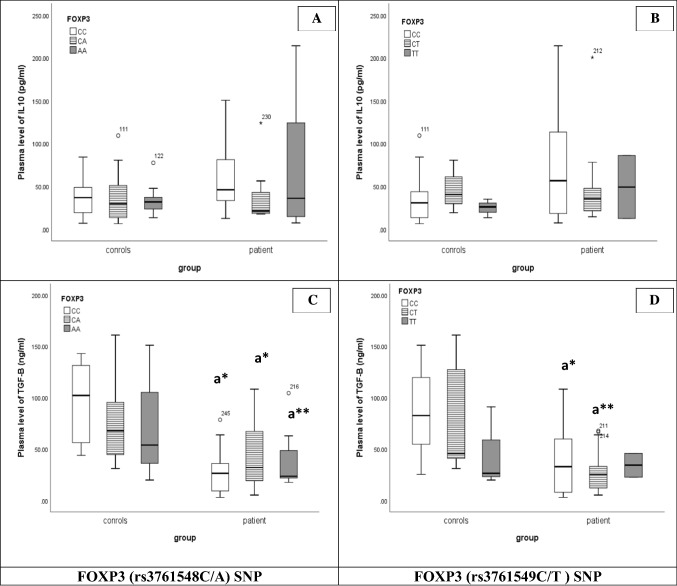
Association between FOXP3 SNPs [rs3761548C/A (**A**, **C**) and rs3761549C/T (**B**, **D**) and Treg related cytokine (IL-10 & TGF-β; respectively) in controls and patients. Line inside the box representing Median, Boxes representing 25th and 75th percentiles and the lines outside the boxes indicating 10th and 90th percentile. a: relative to control; *:p < 0.05/NS, **: p < 0.01/p < 0.05 (p/pc)

To study the impact of these SNPs on cytokine expression for Treg and Th17 cells, we compared the expression level of related cytokines with each SNP. For Tregs, analysis of *FOXP3* (rs3761548C/A and rs3761549C/T) SNPs with IL-10 plasma levels (Fig. 3A, B) pointed to an insignificant increase of IL-10 levels in all genotype distribution in the patient group compared to the control group. Concerning TGF-β, a decrease in TGF-β level in *FOXP3*rs3761548CC (p < 0.05/NS), CA (p < 0.05/NS), AA (p < 0.01/p < 0.05), rs3761549CC (p < 0.05/NS) and rs3761549CT (p < 0.01/p < 0.05) genotypes were found (Fig. 3C, D). Thus, no genotype of *FOXP3*rs3761548C/A and rs3761549C/T was responsible for the increase of IL-10 or the reduction of TGF-β in ALL patients.

**Fig. 4:**
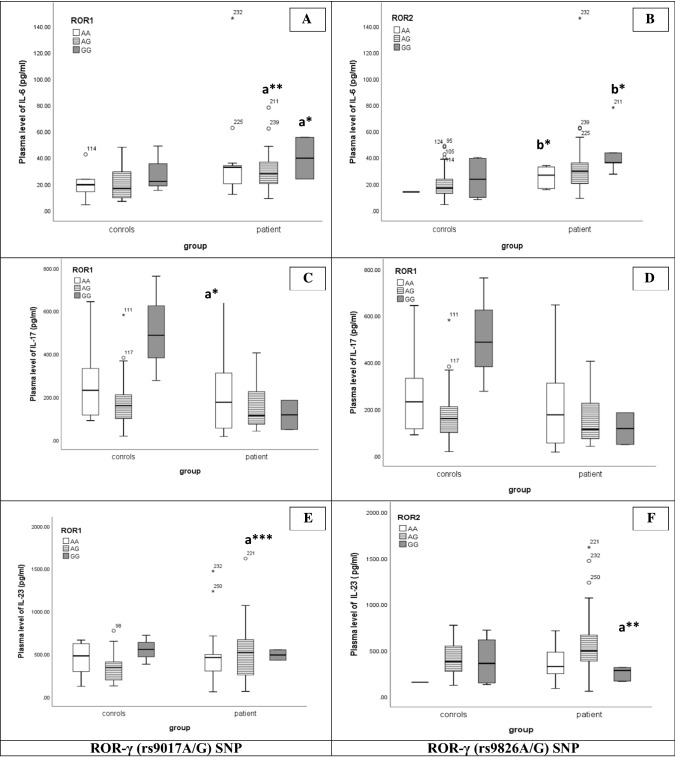
Association between ROR-γ [rs9017A/G (**A**, **C**, **E**) and rs9826A/G (**B**, **D**, **F**)] SNPs and Th17 related cytokine (IL-6, IL-17&IL-23; respectively) in controls and patients. Line inside the box representing Median, Boxes representing 25th and 75th percentiles and the lines outside the boxes indicating 10th and 90th percentile. a: relative to control; a: relative to control [*p < 0.05/NS, **p < 0.01/p < 0.05, ***p < 0.001/p < 0.01], b:relative to control [*p < 0.05/p < 0.05], (p/pc)

Concerning cytokines associated with Th17 cells, IL-6 plasma levels showed a gradual increase with all genotypes of *ROR-γ* (rs9017A/G and rs9826A/G) SNPs in ALL patients compared to controls. An increase in IL-6 level in *ROR-γ* rs9017AG (p < 0.01/p < 0.05) and *ROR-γ* rs9017GG (p < 0.05/NS), *ROR-γ* rs9826 AA (p < 0.05/p < 0.05), *ROR-γ* rs9826 GG (p < 0.05/p < 0.05) genotypes was demonstrated (Fig. 4A, B). Concerning IL-17 levels, a gradual reduction in IL-17 levels (Fig. 4C, D) in all genotypes of *ROR-γ* (rs9017A/G) SNP with a significant reduction (p < 0.05/NS) in GG genotypes in the patient group compared to the control group. *ROR-γ* rs9826AG showed the maximum increase in IL-17 levels in patients compared to controls versus AA and GG genotypes, with no statistical difference between both groups. Different expressions of IL-23 level among *ROR-γ* (rs9017A/G) genotypes were found (Fig. 4E, F). *ROR-γ* rs9017AG genotype was significantly increased (p < 0.001/p < 0.01) in ALL children compared to healthy subjects. On the other hand, the IL-23 level was gradually increased in all genotypes of *ROR-γ* (rs9826A/G) SNP. In ALL individuals carrying the rs9826 GG genotype, there was a significant increase (p < 0.01/p < 0.05) in IL-23 levels compared to controls. Thus, rs9017AG and rs9826GG genotypes might be associated with the increase in IL-23 plasma levels in ALL patients.

## Discussion

*FOXP3* is a crucial regulator of Treg cells' differentiation and function [24]. *FOXP3* polymorphisms were associated with various disorders, including cancer, and certain SNPs have been reported to have functional impacts on immunological tolerance mediated by Treg cells [25, 26]. The present study investigated the genotype and allelic distribution of *FOXP3*rs3761548C/A SNP. We found a significant reduction in CA genotype frequency and a noticeable elevation in CC genotype in female patients compared to control ones. In agreement with this result, Ghasemi et al. [27] data revealed lower frequencies of rs3761548 CA genotype in female ALL children than controls.

Moreover, our findings suggest that, in pediatric females, CC and AA genotypes could be considered risk factors for ALL. Supporting our results following allogeneic hematopoietic stem cell transplantation, the rs3761548CC genotype has been linked to the risk of hepatic veno-occlusive disease development [26]. Consequently, this SNP was proposed as a candidate indicator for predicting serious complications in pediatric ALL patients following allogeneic hematopoietic stem cell transplantation. Contrary, Ghasemi et al. [27] reported AA genotype as a risk factor for the disease in Iranian patients. Furthermore, data of *FOXP3* rs3761549C/T revealed an insignificant change in genotype distribution between both groups. We found the CT genotype in females, and the C allele in males were disease risk factors. In agreement with our data, Ghasemi et al. [27] recorded no significant variation in the frequency of this SNP between ALL patients and controls, although reporting TT genotype as a risk factor. Contrary to our results, Nam and his colleagues [24] found higher frequencies of rs3761549TT genotype compared to other genotypes in leukemic patients who had allogeneic hematopoietic stem cell transplantation.

The rs3761548 *FOXP3*SNPhas been linked to Tregs function in different conditions [28]. Treg cells produce anti-inflammatory cytokines such as IL-10 and TGF-β, which help to suppress the immune system and promote tolerance [29]. Therefore, evaluating *FOXP3*SNPs and their association with ALL as well as IL-10 and TGF-β levels could predict the disease and assess the status of Treg cells. We observed an insignificant increase of IL-10 levels in all genotype distribution of both *FOXP3* SNPs in the current work. In addition to a significant reduction in TGF-β levels in *FOXP3*rs3761548CC, CA, and AA genotypes, no genotype is responsible for the decrease in TGF-β plasma level. Flauzino et al. [29] reported the same results for IL-10 level in multiple sclerosis South Brazil patients and a noticeable link between*FOXP3* (rs3761548) CA and AA genotypes and level of TGF-β1 than those carrying the CC genotype. Another study evaluated the TGF-β level and its association with *FOXP3*(rs3761548) SNP with recurrent spontaneous abortion in Iranian women and recorded no association between the SNP and TGF-β levels [30].

RORγ had been proposed to be a “master regulator" for Th17 differentiation; however, its deficiency did not completely abolish Th17 cytokine expression [31, 32]. Important variations in the functionality of the RORγt promoter are associated with genetic polymorphisms in this gene[33]. There are few studies investigating polymorphism in *ROR-γ* [17, 18, 25, 33–36].

Results of the present work referred to a significant reduction in rs9017AG genotypes and G allele with a significant increase in AA genotype and A allele in ALL patients compared to controls. Our results also indicated that rs9017AA genotype and A allele could be considered disease risk factors. On the other hand, results of *ROR-γ* (rs9826A/G) pointed to an insignificant change in genotype or allele frequencies between normal controls and ALL patients. In our data, rs9826AG genotype was a risk factor for the disease, where AA and GG genotypes might be protective for pediatric ALL. In line with our data, a protective effect of the rs9826GG genotype was recorded in rheumatoid arthritis Egyptian patients [36]. Both SNPs (rs9826 A/G and rs9017 G/A) were previously described by Paradowska-Gorycka et al. [17] and Gholamipoor et al. [18]. In Polish patients with rheumatoid arthritis, Paradowska-Gorycka et al. [17] reported no substantial variations in the frequency of cases and controls. On the other hand, rs9017 GG genotype and G allele were associated with the increased risk of acute kidney injury development in Iranian patients with no risk for rs9826 SNP [18].

The aberrant synthesis of pro-inflammatory cytokines by T-lymphocytes has been demonstrated as a part of the immunopathogenic pathway for various diseases [37].Multiple cytokines and transcription factors coordinate the differentiation, augmentation, and stabilization of Th17 cells. Th17 differentiation is mediated by TGF-β, IL-6, and IL-1β, whereas its amplification and stabilization are orchestrated by IL-21 and IL-23.Emerging evidence suggests that IL-17, IL-21, and IL-22, pro-inflammatory cytokines produced by Th17 cells, may promote immuno-inflammatory reactions in a wide range of diseases with immunological underpinnings [38].IL-17, IL-21, and IL-22, pro-inflammatory cytokines generated by Th17 cells, can promote immuno-inflammatory reactions in conditions with immunological cells, may is essential to determine the status of cytokines belonging to the Th17 cells and the impact of *ROR-γ* on the Th17 pathway in ALL. In this work, we record a notable increase in IL-23 level with rs9017AG and rs9826GG genotypes. Gholamipoor et al. [18] reported insignificant interaction between IL-17 level and rs9017 Spinate kidney injury disease.

This study is the first report of *ROR-γ* gene polymorphisms in pediatric ALL that we are aware of. Our preliminary study illuminates the impact of *FOXP3* (rs3761548C/A) and *ROR-γ* (rs9017A/G) gene polymorphisms on the occurrence of ALL in Egyptian children and to the first time we detect a significant interaction between IL-23 and *ROR-γ* rs9017 SNP in pediatric ALL.

The current research has some constraints that should be considered. The number of patients who were included in the study was relatively small. Further research with a larger sample size is needed to validate our findings. Furthermore, since we could not determine whether *FOXP3*/ROR- SNP genotypes contributed to their expression. We highly recommend considering the measurement of *FOXP3* and *ROR-γ* protein expression to investigate the exact impact of these SNPs on protein expression.

## Conclusion

To conclude, our pilot study focuses on the potential role of *FOXP3* (rs3761548C/A) and *ROR-γ* (rs9017A/G) gene polymorphisms and their correlation with ALL occurrence in Egyptian children. Based on our results,*FOXP3*rs3761548CC and AA genotypeand*FOXP3*rs3761549CTgenotypemight be considered disease risk factors. Concerning the *ROR-γ* gene, rs9017AA genotype, rs9017A allele, and *ROR-γ* rs9826AG genotype are deemed risk factors for pediatric ALL in the Egyptian population. Looking at the disease's clinical manifestations, a notable reduction In *FOXP3*rs3761548 CA genotype was found in relapsed patients compared to non-relapsed ones. From the detected cytokines, only *ROR-γ* rs9017AG and rs9826GG genotypes carriers showed a significant increase in IL-23 level. Collectively, the study's results could aid in a better understanding of the *FOXP3*/*ROR-γ* related genetic impact in pediatric ALL patients, which could influence the immunological status of the patients. Larger prospective studies are warranted to confirm our results.

## Data Availability

The datasets used and/or analyzed during the current study are available from the corresponding author on reasonable request.

## Abbreviations

ALL: Acute lymphoblastic leukemia
CI: Confidence intervals
ELISA: Enzyme-linked immunosorbent assay
EDTA: Ethylene-diamine-tetra acetic acid
*FOXP3*: Forkhead box P3
IL: Interleukin
LD: Linkage Disequilibrium
MDR: Multifactor dimensionality reduction
OR: Odds ratio
OS: Overall survival
PCR–RFLP: PCR-based restriction fragment length polymorphism
PCR-SSP: PCR-sequence-specific primer
PCR: Polymerase chain reactions
RORγ or RORc: Retinoic acid receptor-related Orphan Receptor gamma
SNPs: Single nucleotide polymorphisms
TGF-β: Transforming growth factor
